# Supplementary figures and images for: Transcriptomic and metabolomic differences between banana varieties which are resistant or susceptible to Fusarium wilt
Source: PeerJ. 2023 Dec 12;11:e16549. doi: 10.7717/peerj.16549 (PMC10722978; doi:10.7717/peerj.16549)

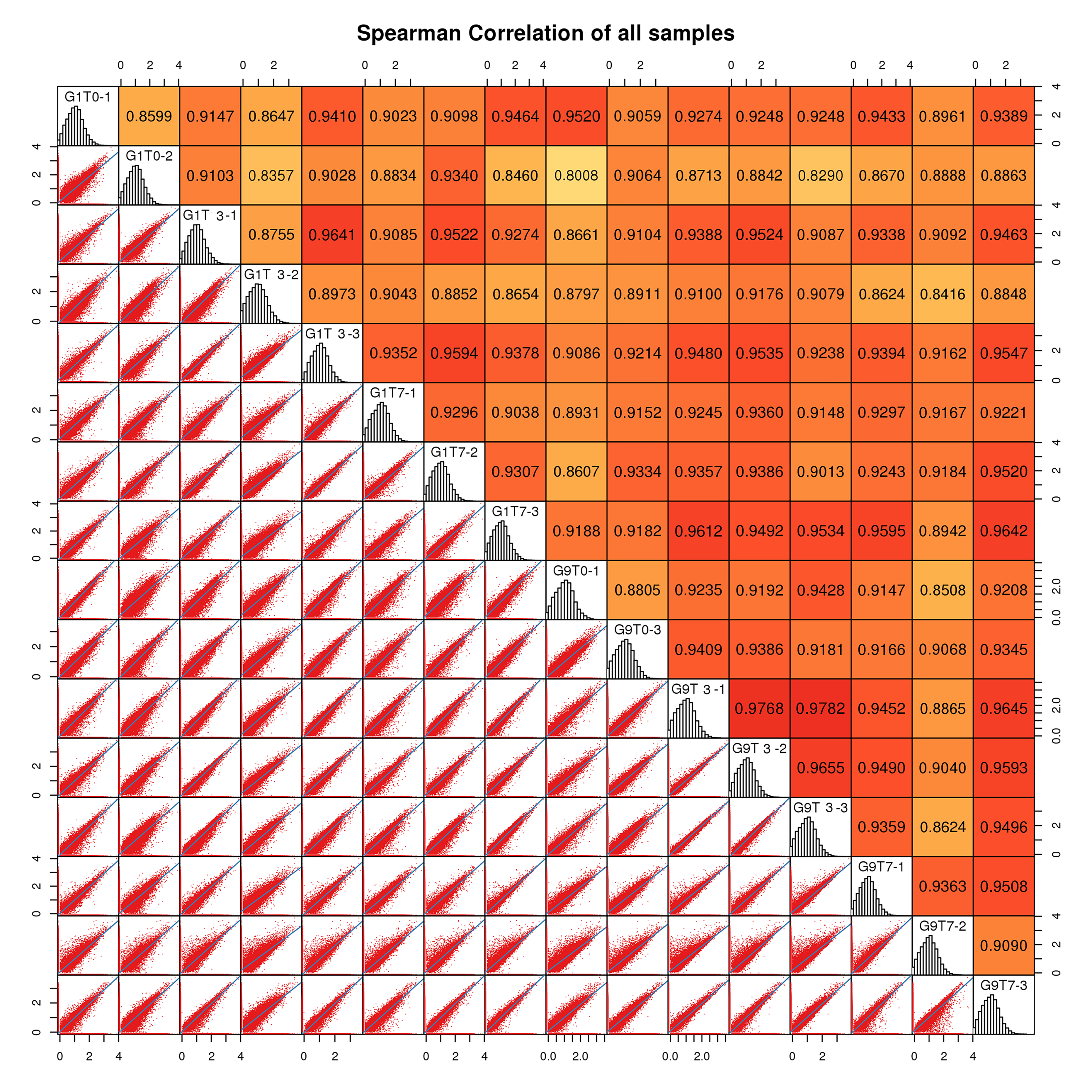

Supplement: Supplemental Information 1 — The spearman correlation analysis was used to test the repeatability of experimental results. [file peerj-11-16549-s001.png]

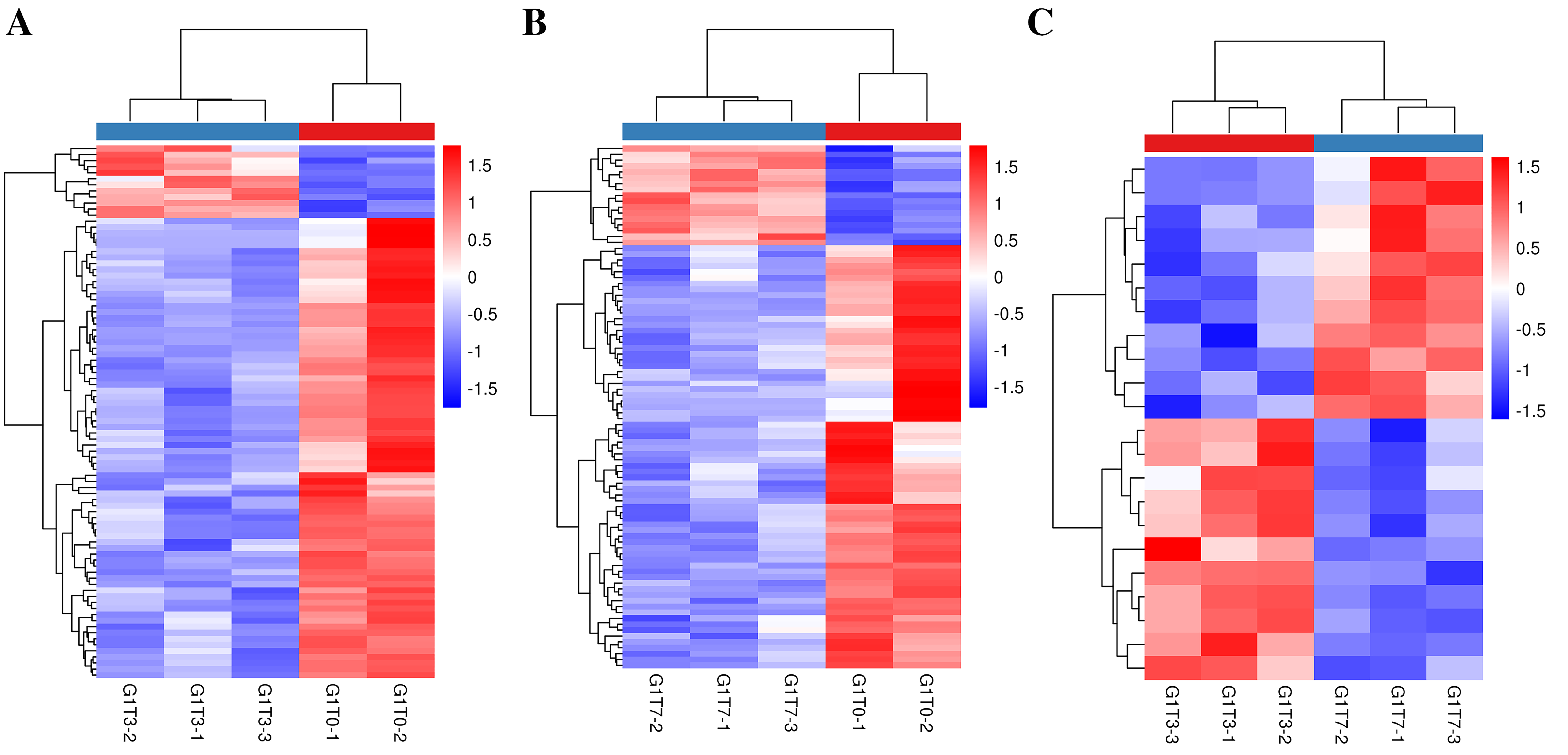

Supplement: Supplemental Information 2 — (A) Heat map of DEGs in G1T3 vs G1T0. (B) Heat map of DEGs in G1T7 vs G1T0. (C) Heat map of DEGs in G1T7 vs G1T3. [file peerj-11-16549-s002.png]

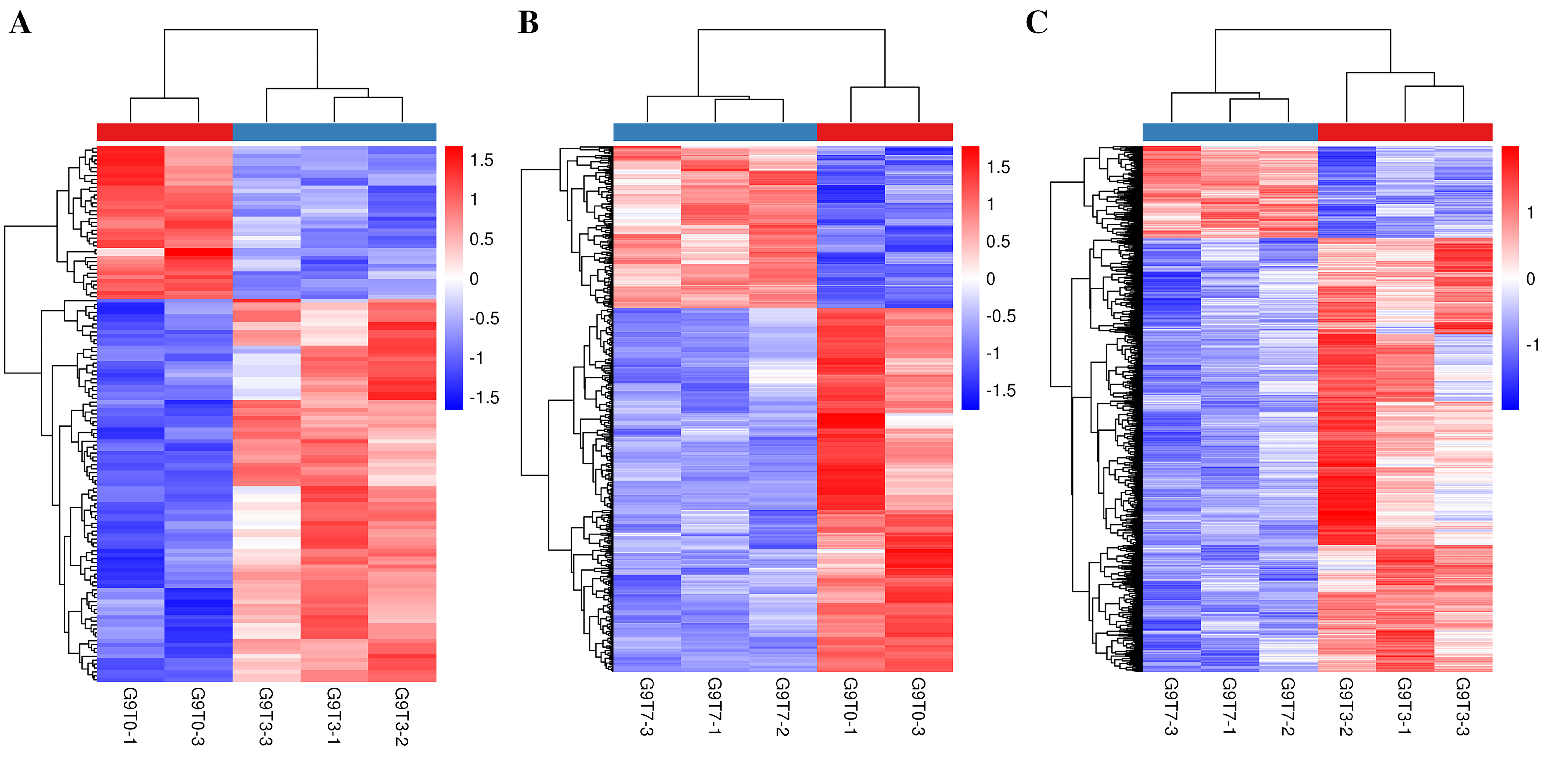

Supplement: Supplemental Information 3 — (A) Heat map of DEGs in G9T3 vs G9T0. (B) Heat map of DEGs in G9T7 vs G9T0. (C) Heat map of DEGs in G9T7 vs G9T3. [file peerj-11-16549-s003.png]

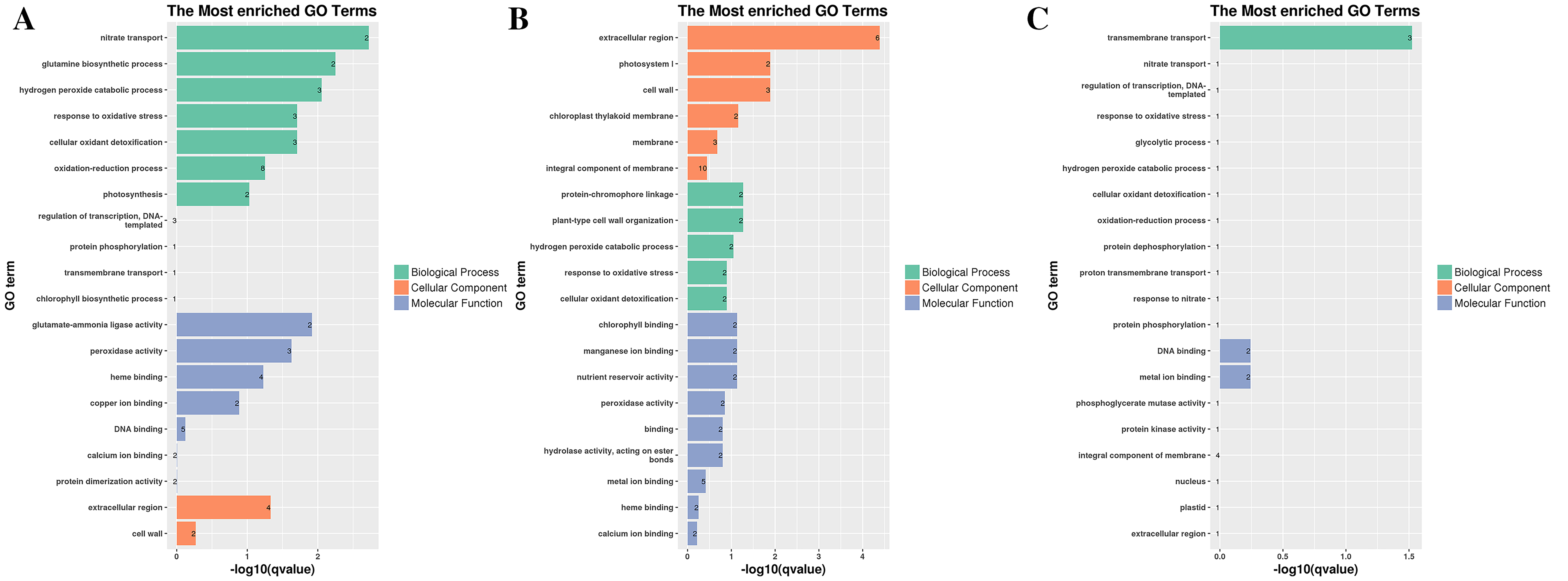

Supplement: Supplemental Information 4 — (A) GO annotation of DEGs in G1T3 vs G1T0. (B) GO annotation of DEGs in G1T7 vs G1T0. (C) GO annotation of DEGs in G1T7 vs G1T3. [file peerj-11-16549-s004.png]

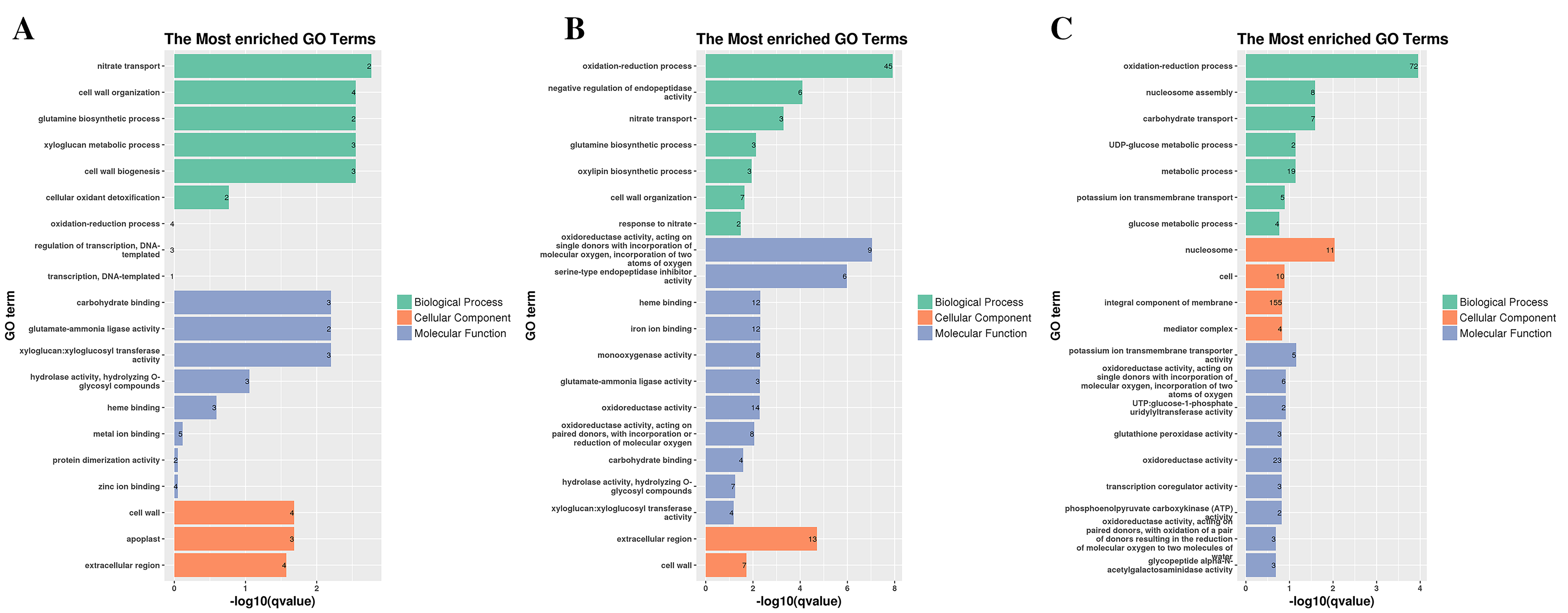

Supplement: Supplemental Information 5 — (A) GO annotation of DEGs in G9T3 vs G9T0. (B) GO annotation of DEGs in G9T7 vs G9T0. (C) GO annotation of DEGs in G9T7 vs G9T3. [file peerj-11-16549-s005.png]

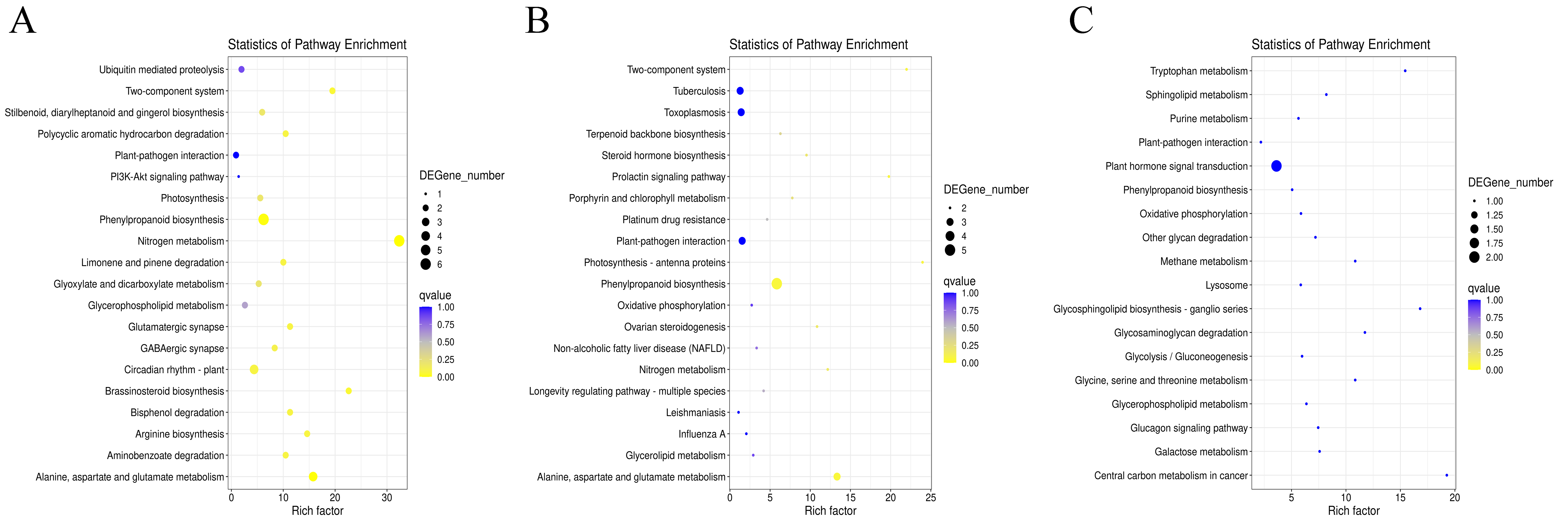

Supplement: Supplemental Information 6 — (A) Most enriched pathways of DEGs in G1T3 vs G1T0. (B) Most enriched pathways of DEGs in G1T7 vs G1T0. (C) Most enriched pathways of DEGs in G1T7 vs G1T3. [file peerj-11-16549-s006.png]

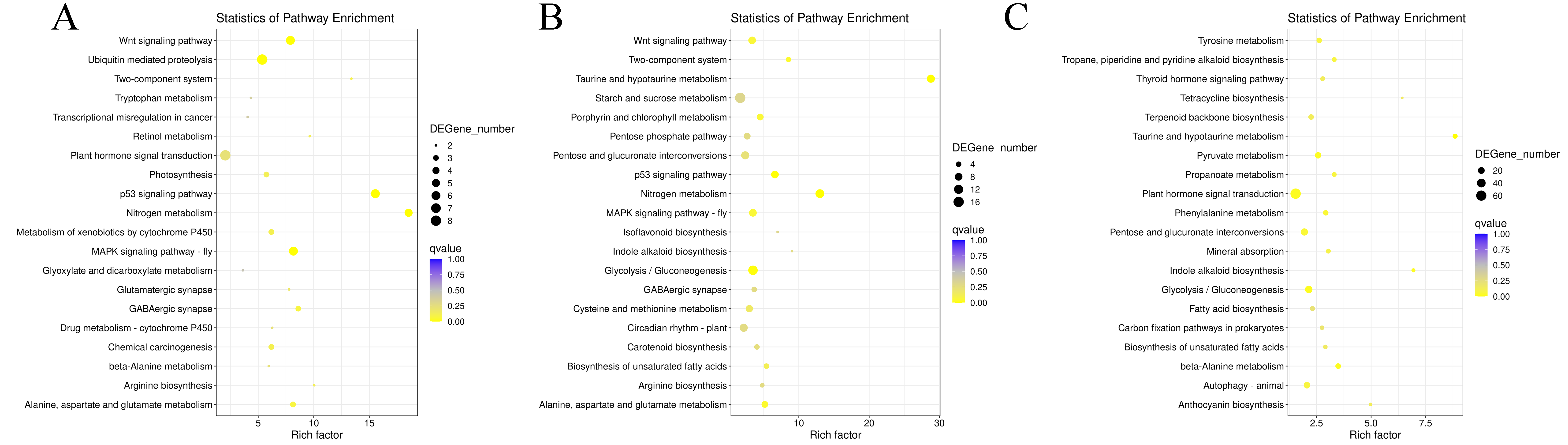

Supplement: Supplemental Information 7 — (A) Most enriched pathways of DEGs in G9T3 vs G9T0. (B) Most enriched pathways of DEGs in G9T7 vs G9T0. (C) Most enriched pathways of DEGs in G9T7 vs G9T3. [file peerj-11-16549-s007.png]

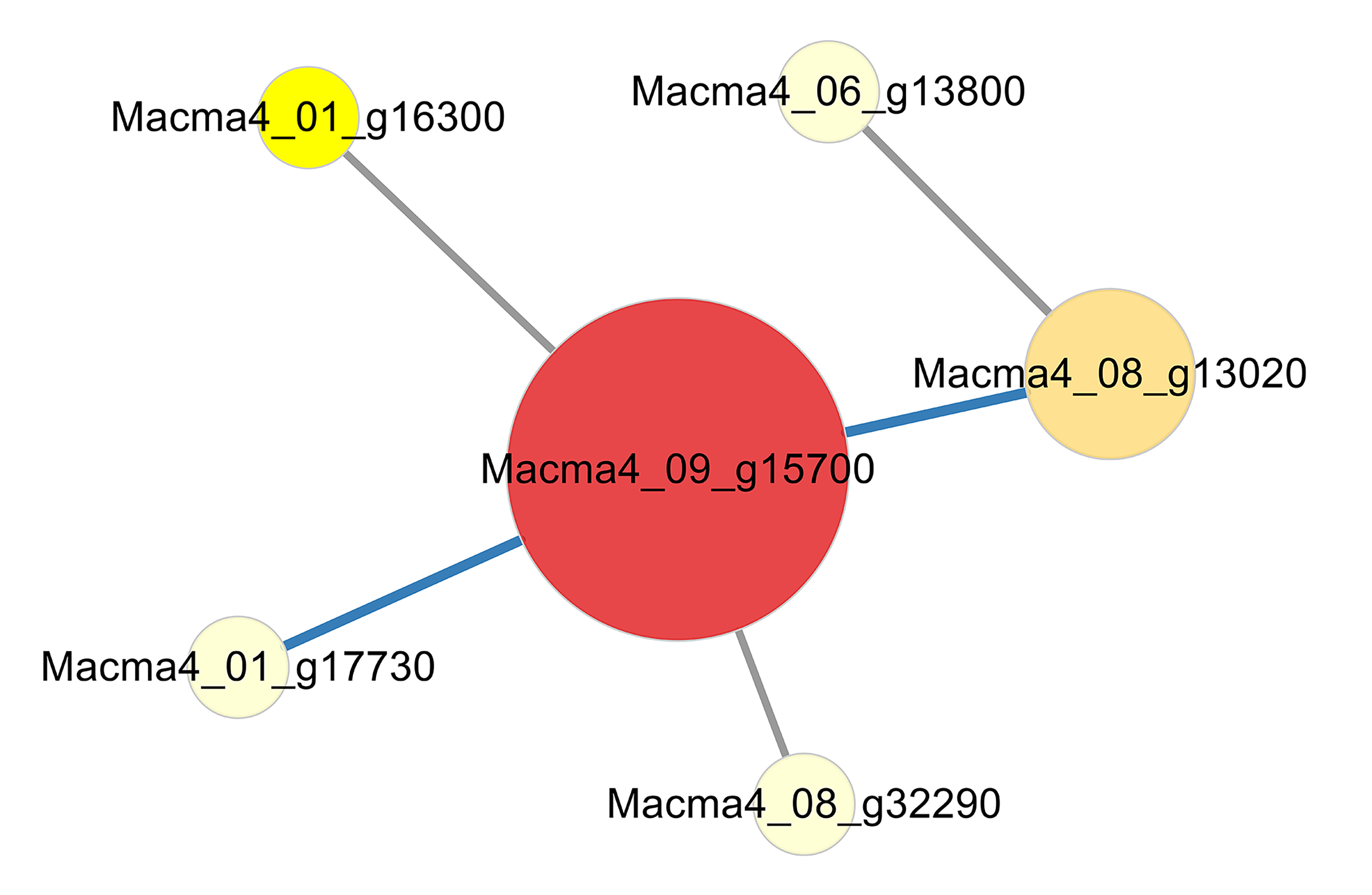

Supplement: Supplemental Information 8 — PPI of DEGs from G1T0 vs G1T3. [file peerj-11-16549-s008.png]

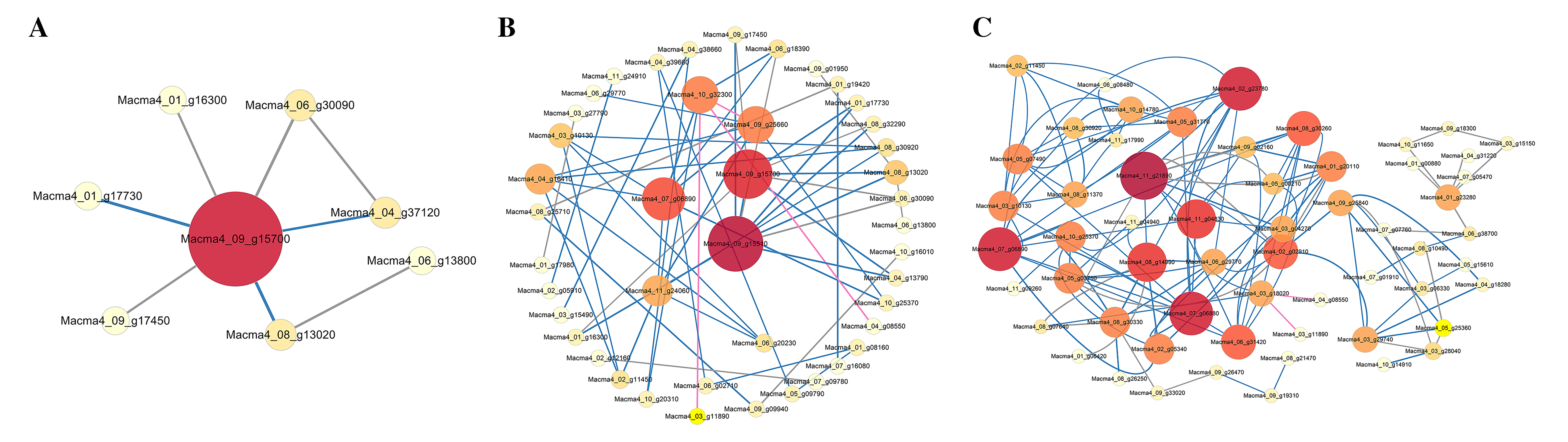

Supplement: Supplemental Information 9 — (A) PPI of DEGs in G9T3 vs G9T0. (B) PPI of DEGs in G9T7 vs G9T0. (C) PPI of DEGs in G9T7 vs G9T3. [file peerj-11-16549-s009.png]

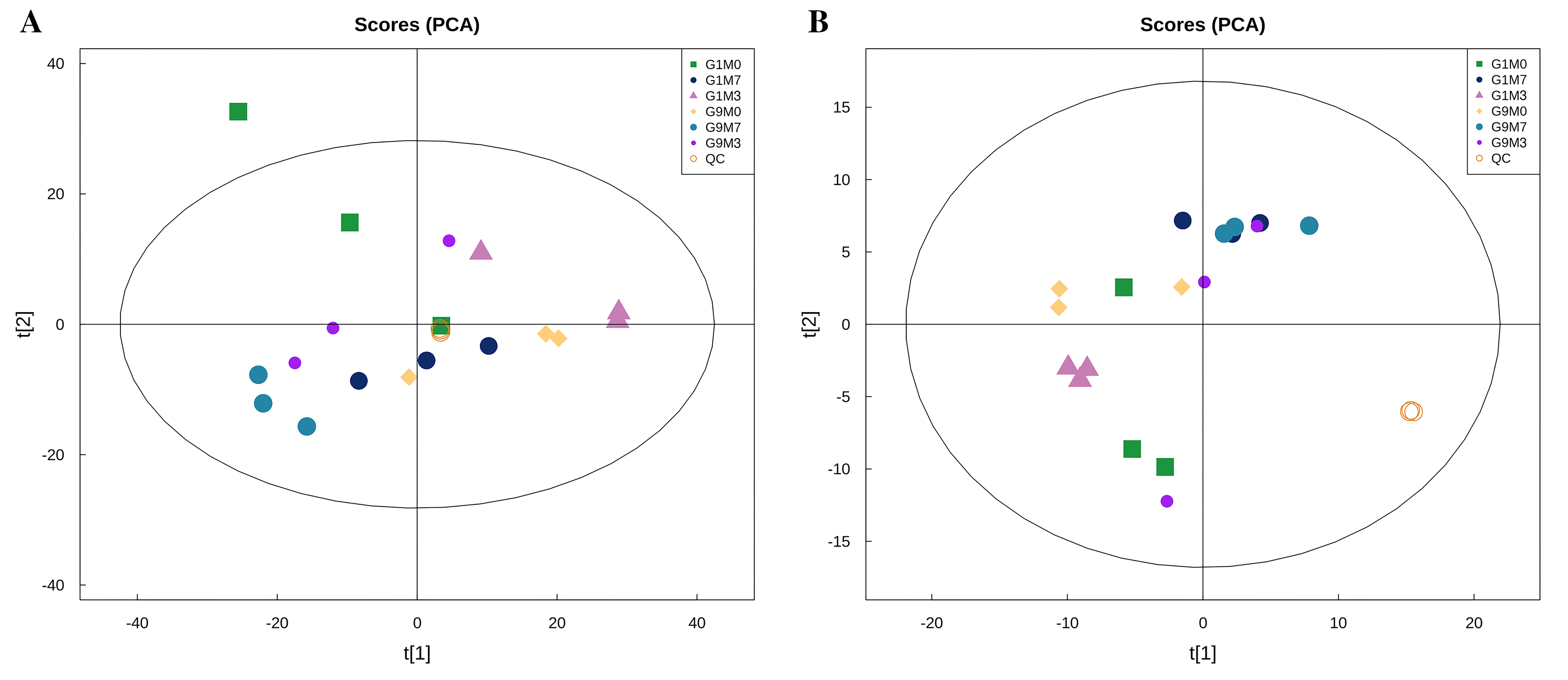

Supplement: Supplemental Information 10 — (A) Negative ion mode. (B) Positive ion mode. T [1] represents principal component 1, and T [2] represents principal component 2. The aggregation degree of QC samples reflects the repeatability of the experiment. [file peerj-11-16549-s010.png]

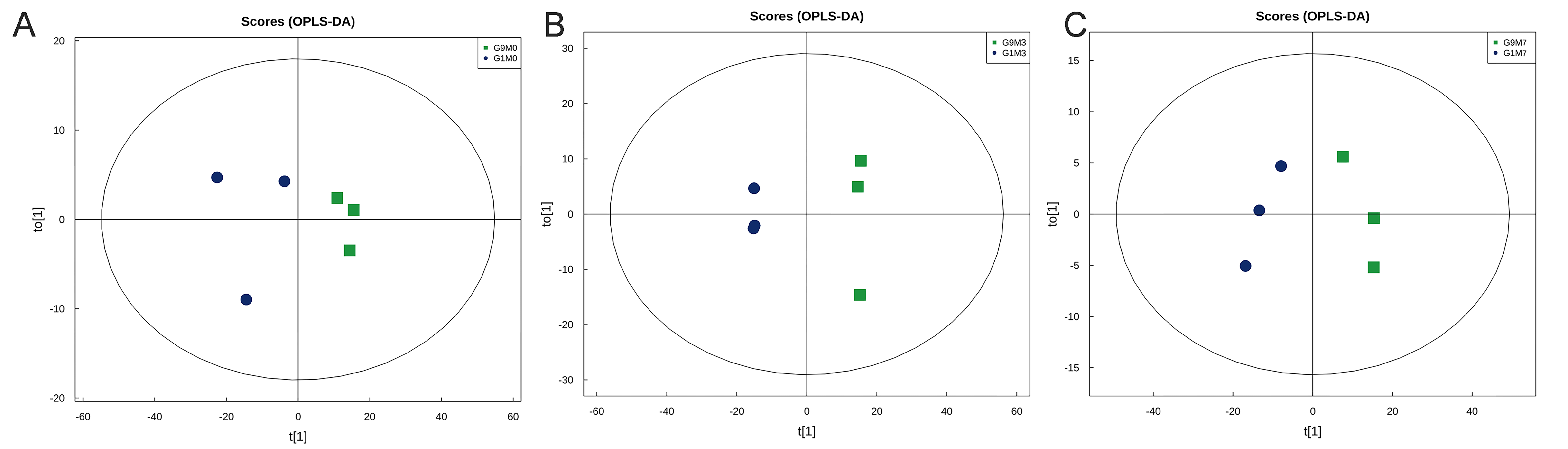

Supplement: Supplemental Information 11 — (A) Negative ion mode. (B) Positive ion mode. T [1] represents principal component 1, and T [2] represents principal component 2. The ellipse represents the 95% confidence interval. Dots of the same color represent biological replicates within the group, and the distribution of dots reflects the differences between and within the group. [file peerj-11-16549-s011.png]

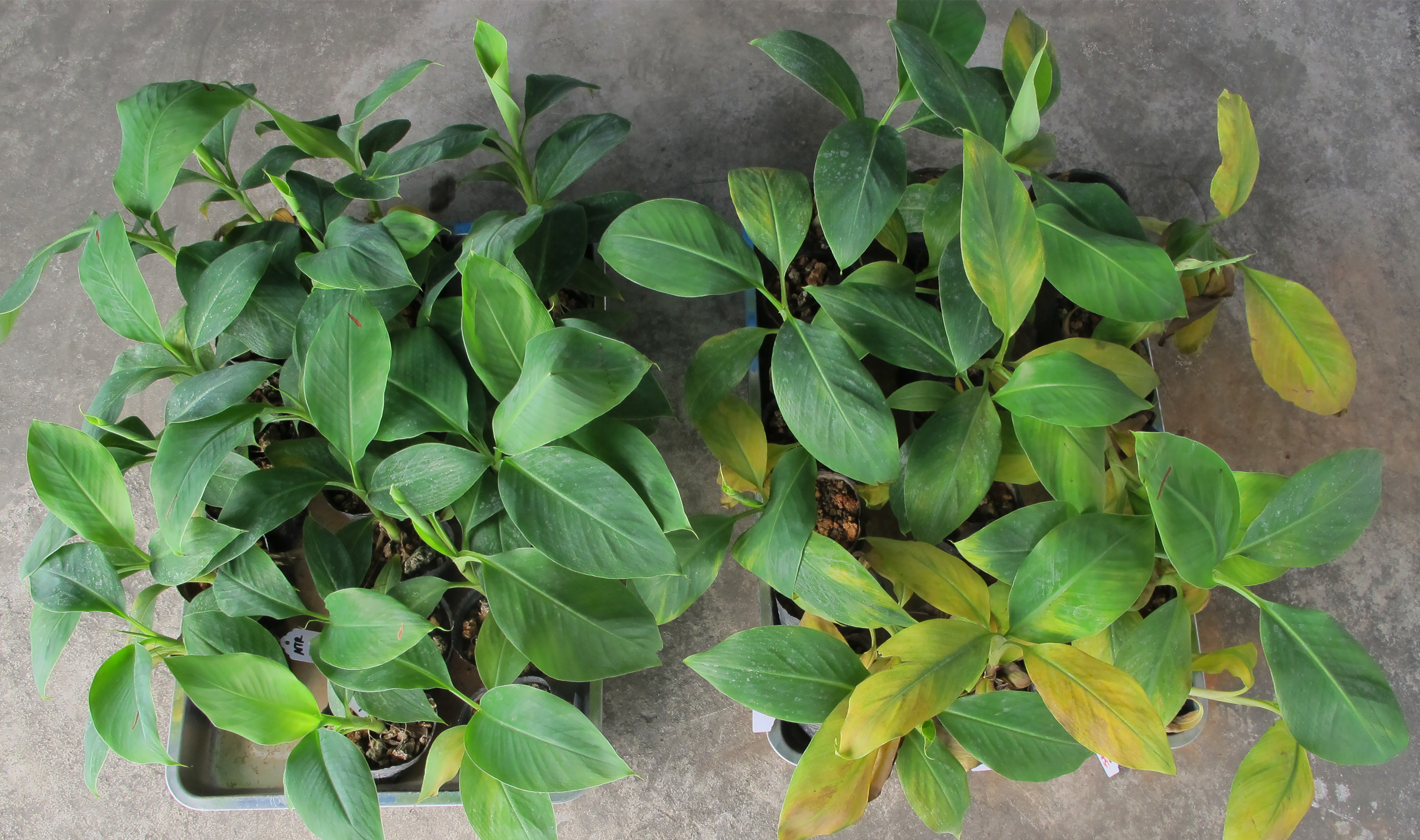

Supplement: Supplemental Information 12 — left was G9 and right is G1. [file peerj-11-16549-s012.png]
